# NGF Eye Administration Recovers the TrkB and Glutamate/GABA Marker Deficit in the Adult Visual Cortex Following Optic Nerve Crush

**DOI:** 10.3390/ijms221810014

**Published:** 2021-09-16

**Authors:** Pamela Rosso, Elena Fico, Louise A. Mesentier-Louro, Viviana Triaca, Alessandro Lambiase, Paolo Rama, Paola Tirassa

**Affiliations:** 1 National Research Council (CNR) of Italy, Institute of Biochemistry & Cell Biology (IBBC), Unit of Translational & Biomolecular Medicine “Rita Levi-Montalcini”, Viale dell’Università 33, 00185 Rome, Italy; pamela.rosso@ibbc.cnr.it (P.R.); elena.fico@ibbc.cnr.it (E.F.); viviana.triaca@cnr.it (V.T.); 2 Department of Ophthalmology, School of Medicine, Stanford University, Stanford, CA 94305, USA; louro@stanford.edu; 3 Department of Sense Organs, University Sapienza of Rome, Viale del Policlinico 155, 00161 Rome, Italy; alessandro.lambiase@uniroma1.it; 4 San Raffaele Hospital, Cornea and Ocular Surface Unit, Eye Repair Lab, Via Olgettina 60, 20132 Milano, Italy; rama.paolo@hsr.it

**Keywords:** BDNF, neurotrophins, optic nerve crush (ONC), synaptic transmission, rat visual cortex (VCx), GABA, glutamate

## Abstract

Eye-drop recombinant human nerve growth factor (ed-rhNGF) has proved to recover the retina and optic nerve damage in animal models, including the unilateral optic nerve crush (ONC), and to improve visual acuity in humans. These data, associated with evidence that ed-rhNGF stimulates the brain derived neurotrophic factor (BDNF) in retina and cortex, suggests that NGF might exert retino-fugal effects by affecting BDNF and its receptor TrkB. To address these questions, their expression and relationship with the GABAergic and glutamatergic transmission markers, GAD65 and GAD67, vesicular inhibitory amino acid transporter (VGAT), and vesicular glutamate transporters 1 and 2 (VGLUT-1 and VGLUT-2) were investigated in adult ONC rats contralateral and ipsilateral visual cortex (VCx). Ed-rhNGF recovers the ONC-induced alteration of GABAergic and glutamatergic markers in contralateral VCx, induces an upregulation of TrkB, which is positively correlated with BDNF precursor (proBDNF) decrease in both VCx sides, and strongly enhances TrkB+ cell soma and neuronal endings surrounded by GAD65 immuno-reactive afferents. These findings contribute to enlarging the knowledge on the mechanism of actions and cellular targets of exogenously administrated NGF, and suggest that ed-rhNGF might act by potentiating the activity-dependent TrkB expression in GAD+ cells in VCx following retina damage and/or ONC.

## 1. Introduction

The neurotrophin nerve growth factor (NGF), as well as its family-related brain-derived neurotrophic factor (BDNF) are considered fundamental during the development of the nervous system and to maintaining the functional integrity of the nervous tissue in adults. Neurotrophins, by interacting with their tyrosinchinase selective receptors (TrkA and TrkB), regulate the survival of mature and precursor neuronal cells, modulate the neuronal activity, and contribute to the structural and functional recovery after injury or diseases [1,2,3,4,5]. As part of the nervous system, the retina and the primary visual areas are also neurotrophin targets [6]. Visual functions in both development and adult life depend on the neurotrophin availability of NGF and BDNF, which are produced in the retina/brain and retrogradely and anterogradely transported by the optic nerve [7,8,9]. 

As those endogenously produced, exogenous NGF and BDNF exert their biological actions on the ocular tissues, including the retina, when ocularly administrated [2,10]. Pharmacological studies demonstrate that NGF applied as eye-drops (ed-NGF) can reach the posterior segment of the eye [7,11,12] and counteract the loss of retinal cells in animal model of retinopathy [13]. Neuroprotective and regenerative effects of ed-NGF on the retina and optic nerve have also been demonstrated in adult rats with unilateral optic nerve crush (ONC), a well-described model of optic neuropathy [14,15,16]. By affecting both anterograde and retrograde transport through the optic nerve, ONC is characterized by a neurotrophin support deprivation, which results not only in retina damage [15] but also in a diffuse degeneration in the primary visual areas contralateral to the lesioned nerve, although effects on the ipsilateral side are also reported [17,18,19,20,21,22,23]. Time course studies showed that ONC-induced structural and functional alterations, both in terms of neurotransmitters and neurotrophic factors, are already observable in the retina [15] and the primary visual area of the lateral geniculate nucleus (LGN) [24] at 24 hrs post-crush, and propagate at cortical level in the following days. Reduced phosphorylation of AKT, increased caspase-3 [24], and cell loss are also found in contralateral visual cortex (VCx) starting from the first week after ONC proving that the activation of the apoptotic cascade is as one of the early event concurring to visual pathway impairment. A decline of neuronal activity [25], early genes expression [26], and neurotransmitters, such as glutamate and c-aminobutyric acid (GABA) [27], are observable starting from the second week after retina or optic nerve lesion. 

Treatments with eye-drop recombinant human nerve growth factor (ed-rhNGF) start one day post-crush and repeated for the following 14 days beside counteracting the retinal ganglion cell (RGC) loss, and suppress the increased expression of inhibitory factors, such as Nogo-A and Rho-A/Rock, responsible for cone growth collapse and blockade of axonal growth [16]. The optic nerve degeneration and the impairment of transmission and/or neurotrophic signals to the brain resulted in an obstacle, suggesting that retinofugal effects might also be exerted by ed-NGF [16]. The findings that ed-NGF improves visual acuity and electro functional parameters and induces prolonged neuroprotection, which results in stabilization/improvement of visual function even after short treatment times in humans [3,28], further indicates involvement of VCx. 

Previous findings showed that ed-NGF increases c-fos expression in brain areas, including the cortex in healthy rats [29], and upregulate the retina and cortical BDNF and TrkB expression [2] which in turn are involved in synaptic plasticity and neuronal network rearranging [30]. The possibility that ed-NGF neuroprotective and reparative effects might extend from the retina to the visual cortex remains to be fully investigated. 

Glutamate and GABA are involved in many neuronal processes of the excitatory and inhibitory synaptic transmission in central nervous system [31,32]. Cortical glutamate and GABA transmission in VCx are considered as markers of visual functions [33,34,35]. 

Glutamate is the prevalent excitatory neurotransmitter in the CNS and glutamatergic transmission is crucial for neuronal activity [36,37,38]. Alterations of the vesicular glutamate transporters 1 and 2 (VGLUT-1 and VGLUT-2) and vesicular inhibitory amino acid transporter (VGAT) in VCx are reported as models of deafferentation, such as ocular enucleation, and monocular deprivation [39,40]. The effects of ed-rhNGF on expression of VGLUT-1, VGLUT-2, and VGAT in the contralateral and ipsilateral visual cortex of adult rats that underwent ONC were analyzed.

Further, a growing body of evidence shows that BDNF and GABAergic inhibition are key mediators of experience-dependent plasticity in the VCx [41], and BDNF, through its receptor TrkB expressed by GABA neurons, regulates cortical plasticity during critical development periods, as well as following visual stimulation or ocular deprivation in adults [42]. 

Thus, for a better characterization of the ed-rhNGF effects, the expression of BDNF and its receptor TrkB, and their relationship with the GABAergic markers, such as the two forms of the GABA synthetizing enzyme glutamic acid decarboxylase (GAD65 and GAD67) were also investigated in the contralateral and ipsilateral VCx of adult ONC rats.

In accordance with previous findings that ed-NGF from the eyes reaches the brain and stimulates recovery of injured-induced structural and molecular changes in cortex [2], the present study indicates that ed-rhNGF might act by potentiating the activity-dependent TrkB expression in GAD positive cells in VCx cortex. 

## 2. Results

### 2.1. Effect of ed-rhNGF Treatment on VGLUT-1 and VGLUT-2 in Visual Cortex of ONC Rats

**Figure 1:**
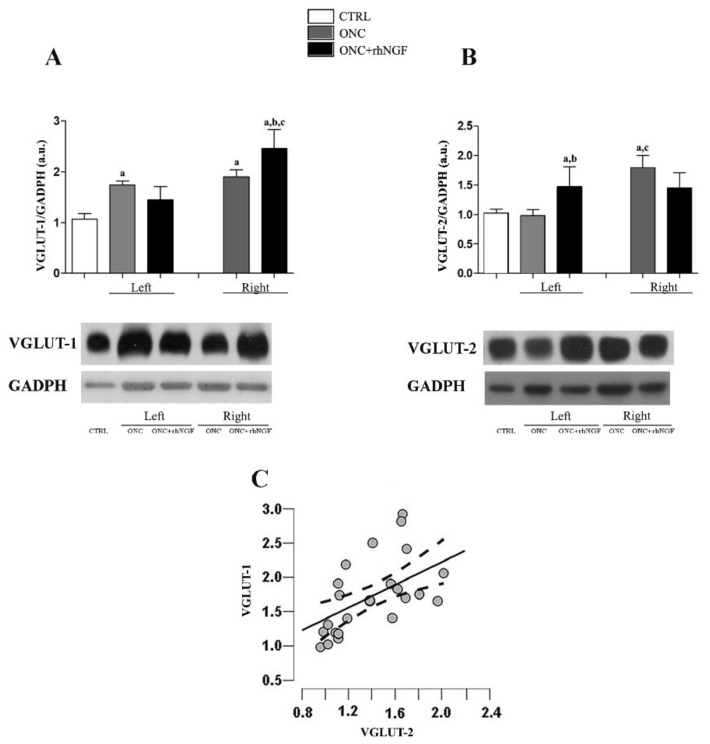
Western Blot of VGLUT-1 and VGLUT-2 level in visual cortex (VCx). (**A**,**B**) shows the semi-quantitative evaluation of levels of VGLUT-1 and VGLUT-2 in the left (ipsilateral) and right (contralateral) VCx of optic nerve crush (ONC) and ONC+rhNGF rats. No changes were detected by comparing the left and right VCx of CTRL rats. Data are expressed as mean optical density arbitrary units (a.u.) and presented as mean ± S.D. (**C**) show the Pearson’s correlation between the VGLUT-1 and VGLUT-2 in the VCx. Statistically different: a vs. CTRL; b vs. ONC; c left vs. right VCx (same treatment condition).

The glutamatergic input in visual structures was investigated in contralateral (right, R) and ipsilateral (left, L) cortex by analyzing the expression levels of VGLUT-1 and VGLUT-2 (Figure 1A–C). 

No differences were found by comparing the levels of glutamate transporters in the two VCx sides in CTRL rats, thus the mean value between the two areas was considered for the comparative analysis.

As it is shown in Figure 1A, VGLUT-1 level was increased on more than 30% in both ONC sides when compared to CTRL (ONC L *p* < 0.001; ONC R *p* < 0.001). A further increase was found in ONC+rhNGF rats where the VGLUT-1 level in the right VCx was about 200% and 140% of the levels detected in CTRL (*p* < 0.001) and ONC rats (*p* < 0.001), respectively. 

The expression level of VGLUT-2 in the right ONC increased with respect to the CTRL rats (*p* < 0.001). Furthermore, VGLUT-2 level in the left ONC resulted different from the the right one (*p* < 0.001). The enhancement of the VGLUT-2 found in the left VCx of ONC+rhNGF was significant versus CTRL (*p* < 0.05). The left ONC+rhNGF, but not the right ONC+rhNGF, increased when compared to ONC VCx (*p* < 0.01) (Figure 1B).

Pearson’s analysis shows a significant positive correlation between the expression levels in the VGLUT-1 and VGLUT-2 in the rat VCx independently of belonging to a particular experimental group (Pearson’s r = 0.557, *p* = 0.001) (Figure 1C).

**Figure 2:**
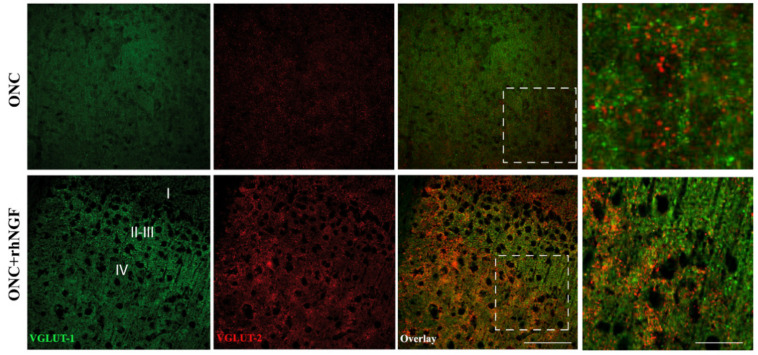
Shows representative VGLUT-1 and VGLUT-2 immunofluorescent staining in the VCx (layers I-IV) of rats. Low magnification of VGLUT-1 (green) and VGLUT-2 (red) in cortical layers I–IV of contralateral visual cortex shows a prevalent VGLUT-1 and a weakly VGLUT-2 staining in cortical varicosities of ONC brains, apparent in the inlet (dotted lines). Ed-rhNGF treatment led to increased positivity of neuronal varicosities to both glutamate transporters in all the ONC cortical layers examined and particularly to VGLUT-1 in layers I–III and VGLUT-2 in layer IV, as more evident in the inlets (scale bars: 200 mm; inlet: 50 mm).

The colocalization analysis by confocal microscopy confirms the WB analysis shows no overlapping between the distribution of VGLUT-1 and VGLUT-2 in the contralateral visual cortex (ONC + rhNGF eye drop treatment) (Figure 2).

### 2.2. Expression of Cortical GABA Markers Following ONC and rhNGF Administration

**Figure 3:**
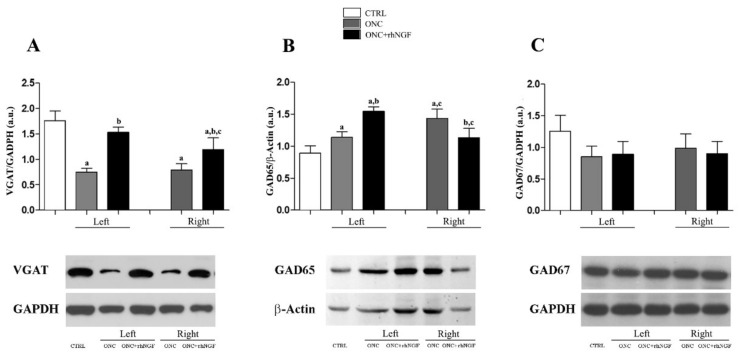
Expression levels of GABA markers in rat VCx. The semi quantitative evaluation of levels of VGAT, GAD65, and GAD67 in VCx of CTRL and left/right VCx of ONC and ONC+rhNGF rats are reported in the graphs (**A**–**C**). Representative cropped gels showing bands corresponding at the three proteins and loading control (GAPDH and β-Actin). Data are expressed as mean optical density (arbitrary units, a.u.) and presented as mean ± S.D. Statistically different: a vs. CTRL; b vs. ONC; c left vs. right VCx (same treatment condition).

The effects of ONC and NGF treatment on the GABAergic transmission was investigated by analyzing the expression levels of VGAT and the two isoforms—GAD65 and GAD67 (Figure 3). No differences in the expression of GABA markers were found by analyzing the left and right VCx of CTRL rats; the mean values between the two VCx sides were therefore used in comparative analysis.

The two-way ANOVA analysis shows significant effects of the groups (CTRL, ONC, and rhNGF treatment), while right and left VCx factors (F_2,24_ = 5.727, *p* = 0.009) and the post-hoc analysis reveals specific differences. A similar decrease of VGAT levels was found in the left and right VCx of ONC rats when compared to CTRL (*p* < 0.001) and ONC+rhNGF rats (*p* < 0.001). Compared to CTRL, the VGAT decreased significantly in the right (*p* < 0.001) but not in the left VCx (Figure 3A).

As far as the expression of GAD65 is concerned, a different effect of ONC and NGF was found in the left and right VCx. Indeed, while GAD65 was unchanged and increased in the left VCx of ONC and ONC+rhNGF, respectively, the opposite trend was found in the right VCx (Figure 3B). The post-hoc analysis confirms the significant difference between the left and right VCx of both ONC groups (*p* < 0.001) and the effects of rhNGF treatment in the left VCx (*p* < 0.001). 

No significant variations of GAD67 were found by comparing the groups and the right and left VCx (Figure 3C).

### 2.3. Effects of ONC and rhNGF Treatment on BDNF 

Since synaptic transmission in the cortex is modulated by BDNF, the effects of ONC and ed-rhNGF treatment on concentration levels of BDNF measured by ELISA and WB analysis of the proBDNF expression were investigated. 

No differences in the expression of BDNF and proBDNF were found by analyzing the left and right VCx of CTRL rats; the means values between the two VCx sides were therefore used in the comparative analysis. 

**Figure 4:**
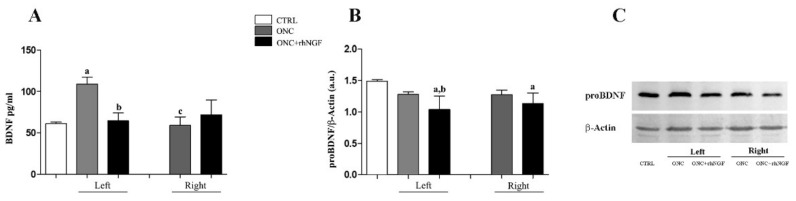
Effecs of ONC and rhNGF treatment on BDNF. BDNF concentration measured by ELISA and the results of WB analysis of proBDNF in VCx of CTRL, ONC, and ONC+rhNGF are shown in graphs (**A**,**B**), respectively. BDNF is expressed as pg/mL protein, and proBDNF is expressed as optical density (arbitrary units, a.u.). A representative gel for proBDNF is reported in (**C**). Data are presented as mean ± S.D. Statistically different: a vs. CTRL; b vs. ONC; c left vs. right VCx (same treatment condition).

The BDNF concentrations measured in the VCx is shown in Figure 4A. The ANOVA and the following post-hoc analysis reveal that BDNF levels were increased in the left ONC VCx (F_2,24_ = 21.606; *p* < 0.001), while they were indistinguishable from CTRL values in the right VCx (*p* = 0.999). In ONC+rhNGF, the concentration of BDNF measured in the left (*p* = 0.998) and right (*p* = 0.800) VCx were similar to CTRL and therefore decreased significantly with respect to the left VCx ONC (*p* < 0.001).

The expression levels of proBDNF in the rats VCx is shown in Figure 4B,C. A significant variation was found in the left VCx of ONC+rhNGF rats when compared to CTRL (*p* < 0.01) and to ONC left (*p* < 0.05), while the right VCx of ONC+rhNGF group was significant only with respect CTRL group (*p* < 0.05) (Figure 4B,C). 

### 2.4. Expression of TrkB in VCx 

**Figure 5:**
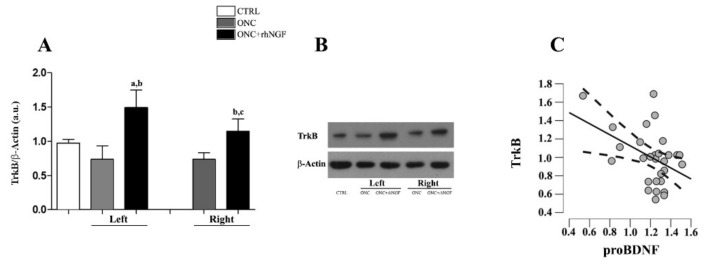
Effects of ONC and treatment with ed-NGF on the expression levels of TrkB in the VCx. WB analysis of TrkB is shown in graph. Data are expressed as mean optical density (arbitrary units, a.u.) and presented as mean ± S.D (**A**). Representative cropped gels showing bands corresponding at the 140 kDa band of TrkB and a loading control (β-Actin) are presented in (**B**). The graph in (**C**) shows the correlation between the expression levels of proBDNF and TrkB in rat VCx. Statistically different: a vs. CTRL; b vs. ONC; c left vs. right VCx (same treatment condition).

The WB analysis shows no differences between the TrkB expression levels in the right and the left cortex in ONC groups (Figure 5A,B), but the trend of TrkB expression level in the VCx was found to decrease in ONC rats (*p* = 0.036) when compared to CTRL. The rhNGF-induced increase of TrkB in both the left VCx when compared to CTRL (*p* < 0.01) and ONC rats (*p* < 0.001), while the right VCx was also significant only vs. ONC rats (*p* < 0.01).

The correlation analysis shows that the expression levels of TrkB and the proBDNF are negatively correlated in rat VCx (Pearson’s r = −0.438; *p*-value = 0.016), suggesting that the expression of BDNF receptor is increased when the levels of proBDNF are low (Figure 5C). 

**Figure 6:**
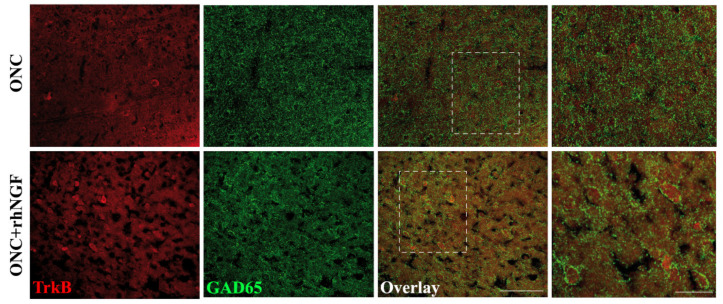
Shows representative TrkB and GAD65 immunofluorescent staining in the VCx (layers I-IV) of rats. Representative TrkB (red) and GAD65 (green) immunofluorescent staining in the contralateral visual cortex of coronal brain sections from ONC+rhNGF or ONC rats. A weak TrkB expression is evident in a minor number of cell bodies and in neuronal varicosities, while a widespread GAD65 staining is present in the ONC VCx. Upon rhNGF treatment, TrkB positive cell soma and neuronal endings are strongly enhanced. Please, note the GAD65 immunoreactive afferents surrounding most of the TrkB positive cell soma in the ONC+rhNGF VCx (inlet, dotted lines). (Scale bars: 200 mm; inlet: 50 mm).

The confocal observation supports the WB data showing an enhanced distribution of TrkB immunofluorescent in the contralateral VCx of ONC+rhNGF rats with respect to ONC condition (Figure 6). Using double immunofluorescence with antibodies against TrkB and GAD65, it was also found that GABA marker immunoreactive afferents surrounding most of the TrkB positive cell soma in the ONC+rhNGF visual cortex, although widespread distribution of GAD65 was also found in ONC cortex. 

## 3. Discussion

Evidence accumulated in the past years supports the neuroprotective effects of exogenous NGF on the ocular tissues of both the anterior and posterior segments of the eye in humans and animals [43]. Using a well-described ONC model to induce a neurotrophin-dependent degeneration of RGCs that propagate the brain retina recipients’ visual areas, including the VCx [15,16], the present study analyzed the effects of ed-rhNGF administration on the VCx during the first two weeks after nerve crush. 

In line with the previous observations that ed-rhNGF is able to counteract ONC-induced RGC loss, and the optic nerve degeneration by obstacle the glia scar formation and the growth inhibition of nerve fiber [16], our present results shows that ed-rhNGF administration contrasts the ONC-induced alteration of the neurotransmitters and neurotrophin markers in VCx. The findings that ed-rhNGF upregulates TrkB both contralateral and ipsilateral VCx, and stimulates VGAT and GAD65 in cortical cells, suggests that NGF obstacles the anterograde degeneration in VCx by an BDNF-mediated action on survival and plasticity.

The glutamate/GABA-network, which represents the principal excitatory/inhibitory system in VCx is regulated by retina input, and therefore variations of neurotransmitter markers, including the vesicular transporters VGLUT-1, VGLUT-2, and VGAT in brain visual areas are considered as pathologic indicators of functional retina and optic nerve [27,34,35,38]. In VCx the localization of the glutamate vesicular transporters also account for their functions: VGLUT-2 is mainly found in thalamo-cortical terminals [44], while VGLUT-1 is highly localized in the intrinsic and cortico-thalamic connections [45]. An overlap distribution and an activity-dependent expression of VGAT and VGLUT-1 are also described in VCx, and contribute to the excitation/inhibition balance in physiological as well as pathological conditions [31,46]. Following unilateral retina deafferentation, a glutamate/GABA deficit is observable in the contralateral VCx, but it is also responsible for the changes of neuronal activity and structural rearranging occurring in the ipsilateral VCx [40].

Congruently with their distribution and response to unilateral deafferentation, our biochemical and immunofluorescent confocal analysis shows that VGLUT-1 and VGLUT-2, as well as GABA markers were differently altered in VCx of ONC and ONC+rhNGF rats. 

We found that the expression of VGLUT-2 in ipsilateral and contralateral VCx was decreased and increased, respectively, in ONC rats, as also found by Sergeeva and colleagues [40] who demonstrated an association between the potentiation of visual response and the VGLUT-2 in ipsilateral VCx, thus indicating an involvement of both hemispheres in the functional and molecular modifications in response to decreased retina input. 

An increase of VGLUT-1 in both VCx sides of ONC rats was also found. Only few data about VGLUT-1 in adult VCx are available. In fact, no changes were observed when comparing the ipsilateral and contralateral VCx after bilateral eye enucleation [44]. However, increased of VGLUT-1 cortico-genicolate projections are demonstrated after retina deafferentation during postnatal development [40]. The strengthening of cortico-cortical [47,48,49], and the plasticity of cortical circuits and cross modal innervation in or near the lesioned area are also observable following visual input loss [50] in humans and animal models, suggesting that the VGLUT-1 levels might reflex the intra-cortical remodeling [51]

In this context, it is worth to mention that the effects of ONC on the ipsilateral and contralateral visual areas in rodents is thought to be strictly associated with the developing regulated segregation of the crossing and non-crossing fibers at prechiasma and chiasma levels [52] A peculiar difference in rodents with respect to humans is that the RGCs axons are mixed, and the direction of fibers might change along the course of the optic tract. During development, the fiber direction and/or re-organization is influenced by the presence of glia, but also by factors regulating axonal growth [6,52], including Nogo-A, a signal inhibiting axonal growth and non-neuronal cell spreading [53].

In vitro and in vivo studies demonstrated that in developing visual system, Nogo plays a role in directing uncrossed axons to the ipsilateral optic tract, and that perturbation of Nogo receptor (NgR1) retina expression might results in alteration of normal axon crossing and non-crossing route at midline chiasma [54,55]. Whether a similar mechanism might occur in adults, and/or following retina deafferentation is still unclear.

In a previous study, we reported that the increased expression of Nogo-R and its downstream signals ROCK increased in the retina a with crushed nerve, and to a lesser extent in the contralateral retina in the first days following unilateral ONC, by preceding the accumulation of Nogo-A at optic nerve crush site [16]. At variance with crush retina, no RGC loss and activation of apoptotic signals were detected in no deafferented retina. Whether this specific pattern of ONC induced modification might affect selected RGCs and/or whether the rate of cross and uncross fibers form crushed eyes was not analyzed by us and needs further investigation. However, it is reasonable to hypothesize that the early events occurring at the retina levels might contribute to the reorganization of fibers at the nerve levels as a part of a compensatory plasticity mechanism in adults. The findings that axons of the RGC cells survived to unilateral ONC in adult rats, which are still in connection with the superior colliculi are subject to rearranging within the optic nerve during the first two weeks after the crush [56], support our hypothesis. It is further possible that both neurodegeneration and compensatory plasticity are contemporaneously present in ONC VCx and that treatment with ed-rhNGF, which has shown to exert neuroprotective, also by contrasting glia activation [2], might act at different levels of the events triggered by interruption of optic nerve signals to brain.

As far as the glutamate/GABA alteration is concerned, our data show that ed-rhNGF induces changes of VGLUT-1 and VGLUT-2 in both contralateral and ipsilateral VCx, respectively, indicating that NGF stimulates glutamate transport at both thalamo-cortical and intra-cortical levels. It is however worth to note that ed-rhNGF results in an abolishment of the ONC-induced decrease of VGAT in both contralateral and ipsilateral ONC VCx, and in the upregulation of GAD65 in ipsilateral ONC VCx, suggesting that GABA network might be the main target of NGF recovery action.

Our confocal microscopy observations might confirm this suggestion by showing that the ed-rhNGF stimulates the increase of VGLUT-1 and VGLUT-2 immunoreactivity mainly in the cortical layer receiving thalamo-cortical projects [57,58], and enhances the expression of GAD65, which is found primarily in presynaptic terminals of inhibitory intra-cortical neurons [59,60]. 

The works by Fattorini et al. [31], which demonstrated that the expression of VGLUT-1 in brain, including cortex, correlates with the VGAT expression, and that VGLUT-1/VGAT might contribute to regulating excitation/inhibition balance in physiological as well as pathological conditions [31] support our data, and led us to speculate that ed-rhNGF might favorite the release of GABA by the cortical interneurons. Our findings on the effects of ONC and treatment with ed-rhNGF on the BDNF/TrkB expression in VCx further support this hypothesis.

BDNF/TrkB is known to regulate VCx activity from development to adult life by modulating GABA inhibition and cortical plasticity [61]. Alterations of BDNF and TrkB expression in VCx in animal models of retina lesion and ocular occlusion result in a decrease of GABA release and GAD65 expression, which are associated with visual impairment and/or a delay of recovery [62,63,64]. 

In line with these observations, we found the ed-rhNGF counteract the ONC-induced elevated levels of proBDNF and strongly stimulate the expression of TrkB in both VCx sides. ONC+rhNGF rat cells expressing VCx are surrounded by intense GAD65 immunoreactivity. This last observation, coupled with the biochemical data on the enhancement of VGAT and GAD65 levels, suggests that treatment with ed-rhNGF stimulates the TrkB-mediated increase of GABA in VCx by modulate presynaptic input.

As mentioned above, an NGF-induced neuroprotective effect on cortical neurons might also explain the increase of TrkB, and in turn the recovery of the BDNF/TrkB mediated unbalance of glutamate/GABA in ONC VCx [5]. 

Recently, You and collaborators showed that ONC in adults is characterized by progressive and time-dependent apoptosis in brain visual areas, including VCx, which is preceded by reduced the phosphorylation of AKT, which is known to mediate the pro-survival, growth, and differentiative effects of neurotrophins by binding their TRK receptors [24].

Increased AKT phosphorylation, associated with activation of TrkB and reduced expression proBDNF have been demonstrated to mediate the neuroprotective and anti-apoptotic effects of ed-NGF in prefrontal cortex in animal model of neurodegeneration [2] suggesting that a similar mechanism might also occur in VCx. 

As we recently proposed, cholinergic subcortical projections take part in the mechanism by which ed-NGF might affect brain and exert its neuroprotective action [3,15]. The evidence that BDNF mediated GABA release by cortical interneurons in VCx is regulated by the cholinergic (ACh) afferents from the basal forebrain [65], and that ed-NGF also stimulates cholinergic system in the brain [66] and in the visual cortex might therefore support the idea that the ascending neuroprotective/modulatory effects of NGF might also involve the cholinergic forebrain neurons, which are primary neurotrophin targets in the brain [66]. Further studies will be necessary to elucidate the contribution of the subcortical area in the effects exerted by ed-NGF in VCx.

## 4. Materials and Methods

### 4.1. Animals and Experimental Design

Adult male Long Evans rats (300–350 g) were obtained from Charles River (Charles River Laboratories Italia s.r.l.) and maintained under controlled temperature and illumination (12:12 h light: dark cycle). Water and food were provided ad libitum. All procedures were approved by the Institutional Animal Care and Use Committee of the San Raffaele Scientific Institute and conformed to National Institutes of health guidelines and the ARVO Statement for the Use of Animals in Ophthalmic and Vision Research Experiments are reported here in compliance with the ARRIVE guidelines. 

All animals were kept untouched for about 2 weeks in their cages to recover the stress from transport and to be habituated to new animal facility conditions. After this acclimatization period, rats were submitted to unilateral optic nerve crush (ONC) and subsequently received treatment with vehicle or ed-rhNGF. The rats were sacrificed at 14 days after crush (dac) to evaluate the effects NGF treatment on the visual cortex of ONC rats by morphological and biochemical technique. See following paragraphs for detailed methodological description.

### 4.2. Unilateral Optic Nerve Crush 

**Figure 7:**
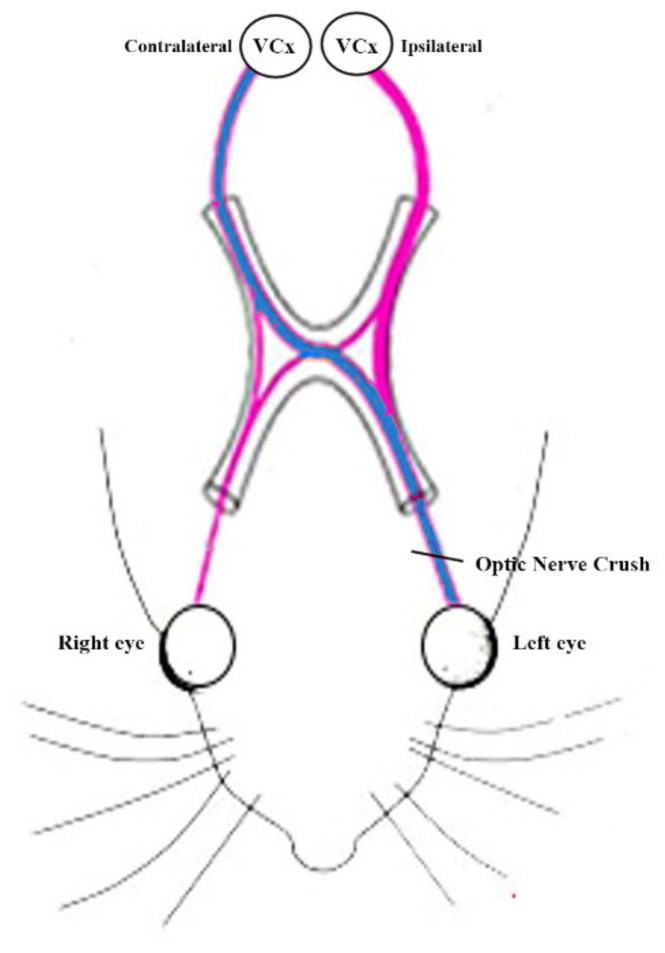
Unilateral optic nerve crush.

The unilateral optic nerve crush was performed as described by [15,16]. The rats were anesthetized with ketamine (70 mg/Kg) and xylazine (10 mg/Kg) given intraperitoneal injection. At the end of the procedure, Oxybuprocaine 0.4% eye drops were used as topical anesthetic and ophthalmic eye ointment was applied to the wound. Under a stereoscopic microscope, the left optic nerve was accessed by an incision in the skin and exposed and the dural sheath surrounding it was cut longitudinally. The left optic nerves were crushed using tweezers (Dumont 5# 45°, 0.05 × 0.01 mm mm tip, World Precision Instruments, Germany). The tweezers were then used to apply pressure on the nerve for 15 s, at 1 mm from the eye. The contralateral right nerves were untouched and served as internal control (Figure 7). After the process, the incision in the skin was sutured and topical antibiotic eye drop (Levofloxacin 5 mg/mL) was applied to the cornea. After treatment, rats were returned to their cages with a heat pad and given a subcutaneous injection of Carprofen (5 mg/Kg) for postoperative analgesia. Rats were monitored for the next four hours to verify the recovery from anesthesia and surgery. Animals showing signs of compromised blood supply were excluded from the study.

As reported in previous studies [15,16], ONC induces a progressive loss of RGCs and induces a deficit of retina input to the brain. At 14 dac, the more dramatic reduction of RGCs was more than 80%, associated with relative astrocytes and GFAP expression in Müller cells. The activation of apoptotic and inflammatory markers in the crushed retina was accompanied by the reduction of TrkA and an increase of p75NTR/proNGF. The RGC axon regrowth ability and the number of fibers beyond the crush site were also affected. The dense accumulation of cells expressing factors inhibiting regrowth and the presence of glia scar further contributed to the morphological changes at the optic nerve level.

### 4.3. NGF Treatment

Based on previous observations on the efficacy of ocular administration of NGF [16,28], rhNGF was used for topical eye drops (ed) administration at the concentration of 540 μg/mL (ed-rhNGF 540). Rats received a droplet of 10 μL of ed-rhNGF or ed-vehicle on the ocular surface of the left eyes (crushed nerves), immediately after crush and then twice a day (early morning and late afternoon) and were euthanized at 14 dac. The same volume of vehicle was administrated on the right eyes as internal control of treatment. 

### 4.4. Brain Dissection and Tissue Lysate Preparation

Fifteen rats (n = 6 ONC, n = 6 ONC + rhNGF, n = 3 untreated rats, CTRL) were deeply anesthetized and sacrificed by cervical dislocation. The brain was removed and the visual cortex contralateral (right) and ipsilateral (left) with respect to the ONC (Figure 7) were quickly dissected on ice and stored at −80 °C until use. To extract proteins, samples were homogenized by ultrasonication in RIPA buffer (50 mM trisHCl, pH 7.4; 150 mM NaCl; 5 mM EDTA; 1% Triton X−100; 0.1% SDS; 0.5% sodium deoxycholate; 1 mM PMSF; 1 mg/mL leupeptin), kept in cold room on rotate shaker for 2 h to allow the complete tissue disaggregation and cell lysis, and then centrifuged at 13,000 rpm for 30 min at 4 °C, to remove tissue debris. The supernatants were used for total protein concentration measured by the Bio-Rad assay. All lysate samples were used for subsequent molecular analyses ELISA and Western Blot (WB) as described below.

### 4.5. BDNF ELISA 

The concentrations of BDNF in rat VCx were measured by ELISA with the BDNF Duoset ELISA (R&D Systems Inc., Minneapolis, MN, USA; DY248), according to the manufacturer’s instructions. The colorimetric reaction product was measured at 450 nm using a microplate reader (Multiscan EX, ThermoFisher, Waltman, MA, USA). The data were expressed as pg/mL protein and presented as means ± SD. 

### 4.6. Western Blot Analysis 

Brain samples prepared as described previously were utilized for WB analysis. Briefly, 20 or 40 µg of total proteins were resolved by 8% or 12% SDS-PAGE at 30 mA (constant current) for about 60–90 min. Protein transfer onto nitrocellulose membrane was carried out by using trans-blot turbo transfer system (Bio-Rad Laboratories, Milan, Italy) for 10 min at room temperature (RT).

The nitrocellulose membranes were incubated for 1 h at RT with blocking buffer constituted by 5% Bovine serum albumin (BSA) or 5% non-fat dry milk in TBS-T (10 mM Tris, pH 7.5, 100 mM NaCl, and 0.1% Tween-20) and washed three times for 10 min each at RT in TBS-T. Samples were incubated, overnight at 4 °C, with the primary antibodies (Table 1).

These steps were followed by incubation for 1 h with horseradish peroxidase-conjugated secondary IgG antibodies (Bio-Rad Laboratories, Segrate, Italy). The nitrocellulose membrane was then reprobed with glyceraldehyde-3-phosphate dehydrogenase (GAPDH) or β-Actin (chosen as housekeeping proteins) (Table 1). Immunoblot analyses were performed using Clarity Western ECL substrate (Bio-Rad Laboratories, Italy) and image acquisition was performed through using enhanced chemoluminescence detection (GE Healthcare, Little Chalfont, UK) and exposure to Amersham Hyperfilm ECL (GE Healthcare) or using iBright™ CL1500 Imaging System (ThermoFisher Scientific, USA). 

Relative levels of immunoreactivity were determined using densitometry and the software ImageJ (National Institutes of Health, Bethesda, MD, USA) for Windows 10. Values are expressed as arbitrary OD units, and the data are presented as means ± SD. 

### 4.7. Confocal Microscopy Studies

At the end of treatment, three ONC, and three ONC+rhNGF rats were deeply anesthetized with an overdose of ketamine and xylazine and perfused through the ascending aorta with 4% paraformaldehyde in 0.1 M-phosphate buffer, pH 7.4. The brain was dissected and post-fixed in 4% paraformaldehyde at 4 °C. Fixed brains were kept in 20% sucrose solution at 4 °C until use.

Imunofluorescence (IF) was performed on 20 μm cryostat (ASI Instruments, Houston, TX, USA) coronal brain sections including the VCx plates 40–45 (Bregma −5.80–6.80 mm; Interaural 3.20–2.20 mm) according to the 4th edition of the Paxinos and Watson rat brain atlas (Paxinos and Watson, 1998). Briefly, the brain sections were washed with PBS 1X and, incubated with a mixture of rabbit anti-VGLUT-1 (Synaptic systems, 1:500) and mouse anti-VGLUT-2 (Millipore, 1:500) or with a mixture of rabbit anti-GAD65 (Spring Bioscience, 1:200) and mouse anti-TrkB antibodies (Santa Cruz, 1:60), primary antibodies, with shaking at 4 °C overnight. On the following day, the sections were washed with PBS 1X and incubated with a mixture of donkey anti-rabbit Alexa488 (Invitrogen A21206, 1:1000) and anti-mouse Alexa546 secondary antibodies (Invitrogen A10036, 1:1000) for 2 h at RT. The brain sections were then washed with PBS 1X and incubated with 4′,6-diamidino-2-phenylindole (DAPI) (10 mg/mL in PBS 1X) for 5 min and washed again. Finally, the sections were mounted on Super Frost microscope slides and covered with mounting medium for conjugated analysis (H-1000; VectaShield, VectorLabs, CA, USA). Images were acquired with an SP5 confocal laser scanning micro-scope (Leica Microsystems, Wetzlar, Germany). Images were acquired by using 920 and 960 lenses, and a pinhole value of 1 Airy unit. The format resolution was 1024 × 1024 pixels, and the acquisition speed was 10 Hz.

### 4.8. Statistical Analysis 

According to methods previously described, statistical analysis was conducted using two-way ANOVA, with groups (CTRL, ONC, and rhNGF treatment), and right and left VCx being the between-subject factors, followed by Tukey’s post-hoc testing. All data are presented as the mean ± S.D. The significance level was set at *p* < 0.05. The correlations between the levels of VCx molecular markers were determined by using the Pearson correlation coefficient. Again, the significance level was set at *p* < 0.05.

## 5. Conclusions

In conclusion, the present study demonstrates that the ocular administration of rhNGF results in a recovery of the ONC-induced Glutamate/GABA unbalance associated with the reduced expression of TrkB in VCx of adult rats. These findings contribute to enlarge the knowledge on the mechanism of actions and the cellular targets of exogenously administrated NGF, and to better understand the events concurring to the recovery of visual functions following retina and optic nerve lesions.

## Figures and Tables

**Table 1:** Antibodies list.

Antigen	Host Species	Dilution	Producer
VGLUT-1	Rabbit	1:5000	Synaptic System, Germany
VGLUT-2	Mouse	1:1000	Millipore, Temecula, CA, USA
VGAT	Rabbit	1:1000	Chemicon International, CA, USA
GAD65	Mouse	1:1000	Santa Cruz Biotechnology, USA
GAD67	Mouse	1:1000	Millipore, Temecula, CA, USA
proBDNF	Mouse	1:1000	Santa Cruz Biotechnology, USA
TrkB	Mouse	1:1000	BD Biosciences, USA
GAPDH	Mouse	1:1000	Santa Cruz Biotechnology, USA
β-Actin+HRP	Mouse	1:5000	Santa Cruz Biotechnology, USA
